# *Borrelia miyamotoi* FbpA and FbpB Are Immunomodulatory Outer Surface Lipoproteins With Distinct Structures and Functions

**DOI:** 10.3389/fimmu.2022.886733

**Published:** 2022-05-27

**Authors:** Charles E. Booth, Alexandra D. Powell-Pierce, Jon T. Skare, Brandon L. Garcia

**Affiliations:** ^1^ Department of Microbiology and Immunology, Brody School of Medicine, East Carolina University, Greenville, NC, United States; ^2^ Department of Microbial Pathogenesis and Immunology, College of Medicine, Texas A&M University, Bryan, TX, United States

**Keywords:** complement C1r, classical pathway of complement, *Borrelia miyamotoi*, complement evasion, spirochete, BBK32

## Abstract

Pathogens that traffic in the blood of their hosts must employ mechanisms to evade the host innate immune system, including the complement cascade. The Lyme disease spirochete, *Borreliella burgdorferi*, has evolved numerous outer membrane lipoproteins that interact directly with host proteins. Compared to Lyme disease-associated spirochetes, relatively little is known about how an emerging tick-borne spirochetal pathogen, *Borrelia miyamotoi*, utilizes surface lipoproteins to interact with a human host. *B. burgdorferi* expresses the multifunctional lipoprotein, BBK32, that inhibits the classical pathway of complement through interaction with the initiating protease C1r, and also interacts with fibronectin using a separate intrinsically disordered domain. *B. miyamotoi* encodes two separate *bbk32* orthologs denoted *fbpA* and *fbpB*; however, the activities of these proteins are unknown. Here, we show that *B. miyamotoi* FbpA binds human fibronectin in a manner similar to *B. burgdorferi* BBK32, whereas FbpB does not. FbpA and FbpB both bind human complement C1r and protect a serum-sensitive *B. burgdorferi* strain from complement-mediated killing, but surprisingly, differ in their ability to recognize activated C1r versus zymogen states of C1r. To better understand the observed differences in C1r recognition and inhibition properties, high-resolution X-ray crystallography structures were solved of the C1r-binding regions of *B. miyamotoi* FbpA and FbpB at 1.9Å and 2.1Å, respectively. Collectively, these data suggest that FbpA and FbpB have partially overlapping functions but are functionally and structurally distinct. The data presented herein enhances our overall understanding of how bloodborne pathogens interact with fibronectin and modulate the complement system.

## Introduction

In the United States, tick-borne relapsing fever (TBRF) is caused by the spirochetes *Borrelia hermsii* and *Borrelia turicatae*. Whereas these spirochetes are vectored by the soft tick of the *Ornithodoros* genus, a related TBRF pathogen, *Borrelia miyamotoi*, is transmitted by ixodid ticks that also vector the agent of Lyme disease (LD), *Borreliella burgdorferi* (1–6). A recent study suggests that *B. miyamotoi* is more widespread in some areas of the U.S. than previously recognized (7). Human infection by *B. miyamotoi* results in overlapping but differing pathology from TBRF, and is known as *B. miyamotoi* disease (BMD) (4, 6). In immunocompetent hosts, BMD presents as a recurrent influenza-like illness that is treatable with doxycycline, ceftriaxone, azithromycin, and potentially amoxicillin (8, 9). Although BMD-, TBRF-, and LD-causing spirochetes are tick-transmitted, their lifestyles within vertebrate hosts differ (10, 11). In the context of vertebrate infection, LD *Borreliella* are thought to survive within the skin and briefly in the bloodstream as a means to disseminate to deeper distal tissues (12–14), while TBRF and BMD *Borrelia* predominantly exist in host blood, indicating a heightened need to evade both soluble and cellular blood-borne immune components (1, 15).

To survive in immunocompetent hosts, TBRF and BMD spirochetes exhibit a robust antigenic variation mechanism *via* a surface-exposed lipoprotein termed variable major protein (Vmp), which allows them to evade clearance from the adaptive immune response (4, 16–18). Each Vmp-associated serotype leads to high bacteremia until a targeted antibody response lowers the relapsing fever *Borrelia* load, after which a new serotype arises, resulting in a “relapse” (19, 20). TBRF symptoms are correlated with the presence of blood-borne spirochetes and the gene conversion event that produces unique Vmp proteins is associated with relapses for TBRF, though less prevalent in BMD (1, 4). In addition to their Vmp-associated immune evasive strategy, *B. miyamotoi* uses other surface lipoproteins to target innate immune mechanisms, including the complement system (21–25). The complement system serves as a first line of defense against invading pathogens. It is initiated through three pathways known as the alternative (AP), lectin (LP), and classical pathway (CP). The CP plays a critical role in elimination of foreign cells and is initiated by antibody-antigen complexes bound by the circulating complement component, C1 (26, 27). Upon activation of C1, proteolytic cleavage of downstream complement components leads to a self-amplifying cascade, resulting in opsonization and phagocytosis of the target cell, modulation of adaptive immunity, neutrophil synergy, and bactericidal activity through formation of the terminal complement complex (TCC), also known as the membrane attack complex (MAC) (27). To avoid destruction of healthy self-cells, host regulators bind complement components, preventing their activation in circulation, such as C1 esterase inhibitor (C1-INH), or on the surface of cells, such as Factor H (FH) (27, 28). A number of bacterial complement inhibitors have been characterized, from inhibitors of complement activation in Gram-positive pathogens, to inhibitors of the MAC in susceptible Gram-negative pathogens, as well as factors that reduce opsonization and phagocytosis by host immune cells (29, 30).

While the complement/complement-evasion axis is better understood for Lyme disease-associated spirochetes (31), recent reports highlight the likely importance of complement evasion by *B. miyamotoi*. CbiA, a surface-exposed FH, C3, C3b, C4b, and C5-binding lipoprotein, inhibits both the CP and AP (21). BOM1093 is a *B. miyamotoi* vitronectin-binding protein that inhibits complement *via* vitronectin’s endogenous TCC-inhibitory activity (25). Additionally, Vlp15/16 and Vlp18 were shown to inhibit the AP, though the mechanism of this activity has not yet been determined (22). The identification of a range of complement inhibitors, despite the relative nascent research into *B. miyamotoi* pathogenesis, is consistent with its blood-borne lifecycle in the vertebrate host.

We have previously shown that the *B. burgdorferi* BBK32 surface-exposed lipoprotein inhibits the CP by directly binding and inhibiting C1r, a serine protease required for activation of the CP (31–34). Separately, we demonstrated that BBK32 provides resistance to a serum-sensitive strain of *B. burgdorferi*, indicating a biologically relevant role for BBK32 in complement resistance (32–34). In addition, BBK32 is multifunctional, also acting as an adhesin to fibronectin and glycosaminoglycans (GAGs) (35–40). Additional work showed that BBK32 is required for optimal *B. burgdorferi* infection (35, 40). Phylogenetic analyses revealed orthologous protein families to BBK32 in both BMD and TBRF spirochetes, designated FbpA, FbpB, and FbpC (41). Both BMD and TBRF spirochetes harbor different sets of Fbp genes, with *Borrelia miyamotoi* encoding only *fbpA* and *fbpB*. In the TBRF pathogen *B. hermsii*, FbpC has been shown to bind fibronectin, however whether *B. miyamotoi* Fbp proteins retain functional similarity to BBK32 has not previously been explored (41, 42). In this study, we characterized the ability of *B. miyamotoi* FbpA and FbpB to bind human fibronectin and the complement protease C1r. Our detailed structure-function investigation reveals that these proteins exhibit differential interactions with both fibronectin and C1r, differences in the three-dimensional structures of their complement inhibitory domains, and demonstrates their ability to protect a serum-sensitive strain of *B. burgdorferi* from serum-mediated killing. Collectively, our data show that Fbp proteins from *B. miyamotoi* harbor partially overlapping, but distinct functions from one another. The novel finding that FbpB specifically recognizes active C1r may inform future efforts to develop therapeutics that are specific for the classical pathway of complement. The need for such complement-directed drugs is highlighted by the association of dysregulated or overactivated complement in important human pathologies (43–45).

## Materials and Methods

### Growth of Bacterial Strains

*Borreliella burgdorferi* B31 strain B314 (46) (**Table 1**) was grown to mid-log phase in BSK-II and 6% normal rabbit serum (Pel-Freez Biologicals, Rogers, AR) under conventional conditions (1% CO_2_, 32°C, pH 7.6). Genetically transformed *B. burgdorferi* strains were grown with kanamycin at 300 μg/mL. *Borrelia miyamotoi* strain FR64b was grown in MKP-F media (24) under the same conditions listed above for *B. burgdorferi. Escherichia coli* strain NEB-5α (New England Biolabs) was grown in Luria broth media under aerobic conditions at 37°C and, as appropriate, with kanamycin at 50 μg/mL. *E. coli* strain BL21 (DE3) (Thermo Fisher) was grown in Terrific Broth (Fisher Bioreagents) with kanamycin (50 μg/mL) at 37°C until an OD_600_ between 0.6-0.8 was achieved, then moved to 16°C overnight for production of recombinant Fbp proteins.

**Table 1:** Bacterial Strains and Plasmid Constructs used in this study.

*E. coli* strains	Description	Reference
NEB 5α	*fhuA2* (*argF*-*lacZ*)U169 *phoA glnV44* ϕ80(*lacZ*)ΔM15 *gyrA96 recA1 relA1 endA1 thi-1 hsdR17*	New England Biolabs
BL21(DE3)	F^–^ *omp*T *hsd*S_B_ (r_B_^–^, m_B_^–^) *gal dcm *(DE3)	ThermoFisher Scientific
***Borreliella burgdorferi* strains**	**Description**	**Reference**
B314	Serum-sensitive, non-infectious *B. burgdorferi* B31 derivative strain lacking all linear plasmids	(46)
B314 pBBE22*luc*	B314 with shuttle vector encoding *bbe22* and *B. burgdorferi* codon optimized *luc* gene under the control of a strong borrelial promoter (P*_flaB_*-*luc*); kan^R^.	(40)
B314 pCD100	B314 with wildtype *bbk32* under control of its native promoter in pBBE22*luc*; kan^R^.	(32)
B314 pAP8	B314 with *B. miyamotoi fbpA* under control of the *B. burgdorferi bbk32* promoter in pBBE22*luc*; kan^R^.	This study
B314 pAP13	B314 with *B. miyamotoi fbpA-R264A-K343A* (*fbpA* DA) under control of the *B. burgdorferi bbk32* promoter in pBBE22*luc*; kan^R^.	This study
B314 pAP11	B314 with *B. miyamotoi fbpB* under control of the *B. burgdorferi bbk32* promoter in pBBE22*luc*; kan^R^.	This study
***Borrelia miyamotoi***	**Description**	**Reference**
FR64b	*Borrelia miyamotoi* isolate originally discovered in Japan and subsequently found in the U.S.	(47)
**Plasmids**		
pBBE22*luc*	Borrelial shuttle vector containing *bbe22* and *B. burgdorferi* codon-optimized *luc* gene under the control of a strong borrelial promoter (P*_flaB_*-*luc*)	(40)
pCD100	Knock in construct of wild-type *bbk32* with its native promoter in pBBE22*luc*.	(32)
pAP8	Knock in construct encoding *B. miyamotoi fbpA* under the control of the *B. burgdorferi bbk32* promoter in pBBE22*luc*.	This study
pAP13	Knock in construct encoding *B. miyamotoi fbpA*-*R264A-K343A* (*fbpA* DA) under the control of the *B. burgdorferi bbk32* promoter in pBBE22*luc*.	This study
pAP11	Knock in construct encoding *B. miyamotoi fbpB* under the control of the *B. burgdorferi bbk32* promoter in pBBE22*luc*.	This study
pT7HMT-BBK32-C	Construct used for overexpression of recombinant BBK32-C.	(32)
pT7HMT-C1r-CCP2-SP	Construct used for overexpression of recombinant C1r-CCP2-SP.	(33)
pT7HMT-FbpA-C	Construct used for overexpression of recombinant FbpA-C.	This study
pT7HMT-FbpB-C	Construct used for overexpression of recombinant FbpB-C.	This study
pT7HMT-FbpB-Δ5C	Construct used for overexpression of recombinant FbpBΔ5-C.	This study
pT7HMT-FbpA DA-C	Construct used for overexpression of recombinant FbpA DA-C (R264A-K343A).	This study
pT7HMT-MBP-FbpA-N	Construct used for overexpression of recombinant MBP fusion protein FbpA-N	This study
pT7HMT-MBP-FbpB-N	Construct used for overexpression of recombinant MBP fusion protein FbpB-N	This study

### Plasmid Constructs

The following constructs for *B. miyamotoi* FR64b *fbpA* (UNIPROT#: AHH06014.1) and *fbpB* (UNIPROT#: AHH05635.1) were subcloned into pT7HMT or a modification of pT7HMT encoding maltose binding protein (MBP) (48). All plasmid constructs are listed in **Table 1**. *E. coli* codon-optimized DNA fragments were purchased from IDT Technologies gBlock Gene Fragment Service or produced using PCR amplification templated from previously cloned constructs using methodology and constructs previously described (32–34, 48): FbpA-C residues 222-371; FbpA-C-R264A-K343A; FbpB-C residues 264-432; FbpB-Δ5C residues 264-427; FbpA-N residues 152-201; and FbpB-N residues 207-248. Oligonucleotides used are listed in **Table 2**. Residue numbering were based on UNIPROT numbering. The expression constructs for *B. burgdorferi* BBK32-C and C1r-CCP2-SP originated from previous studies (32, 33).

**Table 2:** Oligonucleotides used in this report.

Oligonucleotides	5’ to 3’ sequence	Description	Reference
*pncAF*	TATTGGAATTAATAGGCGGTGATG	Oligonucleotide pair used to confirm *bbk32*, *fbpA*, and *fbpB* knock-in constructs	(32)
*lucF*	GAGGGGTTGTATTTGTTGACG		
BmAUSF	CTTTAAAGGAGAGAAAGCATGATAGTTAAAAGTAAATAT	Oligonucleotide pair used to amplify *B. miyamotoi fbpA* for assembly with the *bbk32* promoter and pBBE22*luc*; used to assemble pAP8	This study
BmADSR	TGCATGCCTGCAGGTCGACCTAATACCAAGGACCATCTC		
K32PBmABmBFusionF	GAGGTACCCGGGGATCCGTACTTTGTTCACCCTCTTG	Oligonucleotide pair used to amplify the *bbk32* promoter for assembly with *fbpA* and pBBE22*luc*; used to assemble pAP8	This study
K32PBmAFusionR	ATATTTACTTTTAACTATCATGCTTTCTCTCCTTTAAAG		
BmBUSF	CTTTAAAGGAGAGAAAGCATGATTTTTAAAAATA	Oligonucleotide pair used to amplify *B. miyamotoi fbpB* for assembly with the *bbk32* promoter and pBBE22*luc* used to assemble pAP11	This study
BmBDSR	GCATGCCTGCAGGTCGACTTATCTTGCAACAATACCTG		
K32PBmABmBFusionF	GAGGTACCCGGGGATCCGTACTTTGTTCACCCTCTTG	Oligonucleotide pair used to amplify the *bbk32* promoter for assembly with *fbpB* and pBBE22*luc* used to assemble pAP11	This study
K32PBmBFusionR	TATTTTTAAAAATCATGCTTTCTCTCCTTTAAAG		
BmAR264AF	GGAAATAAGGCTAGACAATCGATACAAGTCTTATCTGG	Oligonucleotide pair used to amplify the R264A fragment to generate a precursor of pAP13	This study
BmAR264AR	TTTAATAGCTCCTTAAGATCTTTCTCAAGTTCTTCTTTTG		
BmAK343AF	CAAAAGAAGAACTTGAGAAAGATCTTAAGGAGCTATTAAA	Oligonucleotide pair used to amplify the K343A fragment of *fbpA* for site directed mutagenesis of *fbpA* K343 used to assemble pAP13	This study
BmAK343AR	TGCATGCCTGCAGGTCGACCTAATACCAAGGACCATCTC		
BmB_D5C_Fwd_BAM	GATGATGGATCCGACAACCAGTACAAATTCAAACT	Oligonucelotide pair used to amplify the FbpB-Δ5C fragment using the pT7HMT-FbpB-C construct template	This study
BmB_D5C_Rev_Not	GATGATGCGGCCGCTTAGGTACGGGTCA		

Expression constructs for *B. miyamotoi fbpA* and *fbpB* were assembled using the NEBBuilderHiFi DNA Assembly Cloning Kit as described in (34). The *B. miyamotoi fbpA* and *fbpB* genes were transcriptionally linked with the *bbk32* promoter from *B. burgdorferi* to promote equal expression in B314 relative to *bbk32*. The R264A and K343A mutations in *fbpA* were constructed sequentially by synthesizing a 194 bp fragment of *fbpA* from nucleotide 918 to 1112 containing the R264A mutation, and then once the R264A construct was obtained, with a 254 bp fragment from nucleotide 1112 to 1366 containing the K343A mutation. Both DNA fragments were synthesized by IDT gBlocks using the native *B. miyamotoi* strain FR64b *fbpA* sequences. The fragments were each assembled into the *fbpA* gene using primers that added overhangs complementing the *fbpA* gene adjacent to the synthesized fragments. These fragments were then assembled into the *fbpA*-expressing vector using NEBuilderHiFi DNA Assembly Cloning Kit. All plasmids were sequenced to ensure the desired mutations were obtained and that no additional mutations were introduced during the assembly process.

### Transformation of *B. burgdorferi*

Transformation of strain B314 with the plasmid constructs pAP8, pAP11, and pAP13 was done as previously described (33, 49).

### Protein Expression and Purification

Recombinant proteins were expressed and purified as in (32, 33). Briefly, after elution of the Ni column using the Elution buffer (20 mM Tris (pH 8.0), 500 mM NaCl, 500 mM Imidazole), the proteins were exchanged into 20 mM Tris (pH 8.0), 500 mM NaCl, 10 mM Imidazole using a Desalting 26/10 column (GE Healthcare). The His-tag was then removed by incubation with the tobacco etch virus (TEV) protease and 5 mM β-mercaptoethanol overnight at room temperature. The TEV digested proteins were separated from the His-tag by passing over a 5 mL HisTrap-FF column on the FPLC with the captured flowthrough further purified using a HiLoad Superdex 75 PG gel filtration column (GE Healthcare). The single peak after gel filtration was examined using SDS-PAGE analysis, then pooled and exchanged into HBS buffer (10 mM HEPES [pH 7.3], 140 mM NaCl). The C1r-CCP2-SP construct was purified according to previously published protocols with an added final gel filtration chromatography step using a HiLoad Superdex 75 PG column (GE Healthcare) and exchanged into HBS buffer (33, 34, 50, 51). Purified full-length complement proteins of both the zymogen and enzymatically active forms of C1r were obtained from Complement Technology, Inc. (Tyler, TX). Purified full-length human fibronectin was purchased from Millipore Sigma. Sequence alignments of the proteins were generated using Clustal alignment server from EMBL-EBI.

### Surface Plasmon Resonance

Surface plasmon resonance (SPR) was performed on a Biacore T200 instrument set to 25°C with a flowrate of 30 μL/min. The running buffer was HBS-T (10 mM HEPES (pH 7.3), 140 mM NaCl, and 0.005% Tween 20) or HBS-T containing 5 mM CaCl_2_. Proteins of interest were immobilized on CMD200 sensor chips (Xantec Bioanalytics) *via* standard amine coupling as before (32–34). Immobilization quantities were as follows: human plasma fibronectin over MBP-FbpA-C (1015.3 RU), MBP-FbpB-C (1074.1 RU); C1r-CCP2-SP over FbpA-C (1061.2 RU), FbpB-C (877.7 RU), FbpA-C-R264A-K343A (FbpA DA-C) (1591.8 RU); C1r zymogen or active C1r over FbpA-C (852.4 RU), FbpB-C (1116.6 RU). C1r-CCP2-SP over Fbps was performed in multi-cycle using a twofold dilution series of the C1r-CCP2-SP ranging from 0-100 nM. Data was obtained in triplicate with each cycle having an association time of 2 min, a dissociation time of 3 min, and three 1 min injections of regeneration buffer (0.1 M Glycine [pH 2.0], 2.5 M NaCl). Equilibrium dissociation constants (*K*_D_) were obtained from the resulting kinetic fits generated by the Biacore T200 Evaluation Software (GE Healthcare). Zymogen or active C1r binding data for FbpA-C and FbpB-C was obtained in single cycle using a fivefold dilution series of either C1r zymogen or active C1r ranging from 0-100 nM. Data was obtained in triplicate with each cycle having an association time of 5 min, a final dissociation time of 1 hour, and two 1 min regeneration injections with regeneration buffer (0.1 M Glycine [pH 2.0], 2.5 M NaCl). Equilibrium dissociation constants (*K*_D_) were obtained from the resulting kinetic fits generated by the Biacore T200 Evaluation Software (GE Healthcare). Human plasma fibronectin was buffer exchanged into HBS-T and then injected over MBP-FbpA-N or MBP-FbpB-N. Data was obtained in multicycle using a twofold dilution series ranging from 0-500 nM. Data was obtained in triplicate with each cycle having an association time of 2 min, a dissociation time of 3 min, and two 1 min injections of regeneration buffer (10 mM CAPS [pH 10.0]). Steady-state affinity fits were performed using Biacore T200 Evaluation Software.

### Surface Plasmon Resonance Competition Experiments

SPR was performed using conditions described for the direct binding experiments. FbpA-C (935.4 RU), FbpB-C (1082.9 RU), and FbpB-Δ5C (1115.4 RU) were immobilized on a new CMD200 sensor chip (Xantec Bioanalytics) *via* standard amine coupling as before (32–34). 25 nM C1r-CCP2-SP alone, or with 25 nM FbpA-C, FbpB-B, FbpA DA-C, or FbpB-Δ5C were injected over the Fbp biosensors. Injections were performed in triplicate with each cycle having an association time of 2 min, a dissociation time of 3 min, and two 1 min injections of regeneration buffer (0.1 M Glycine [pH 2.0], 2.5 M NaCl). The binding response just prior to injection stop was used to determine differences for the ability of the soluble Fbp proteins to compete with immobilized Fbps for C1r-CCP2-SP binding.

### Generation of Polylonal Antibodies Against FbpA and FbpB

Polyclonal antibodies reactive with FbpA and FbpB were generated by separately immunizing female C57BL/6 mice intradermally with 25 µg either FbpA-C or FbpB-C in an equal volume of PBS and TiterMax^®^ Gold Adjuvant (Sigma Aldrich) as outlined (52). Two weeks after the initial immunization, mice were boosted with 25 µg of the same protein. Two weeks after boosting, mice were euthanized and blood was immediately isolated by exsangunation, allowed to clot, and serum removed after low speed centrifugation. Reactivity and specificity of each serum sample to *B. miyamotoi* FbpA and FbpB was evaluated by Western Blot using purified recombinant protein. All animal work was reviewed and approved by the Texas A&M University Institutional Animal Care and Use Committee (protocol number 2019-0422).

### Far Western Overlay Analysis With Full-Length *B. miyamotoi* Proteins

Far Western overlays were carried out as described in (32, 35). Briefly, *B. burgdorferi* strain B314 derivatives were cultured in BSKII media supplemented with 300 μg/mL kanamycin. Cultures were harvested and *B. burgdorferi* whole-cell lysates were generated for B314 strains producing FbpA, FbpB, FbpA DA, or from the pBBE22*luc* vector-only control. 2.5 x10^7^ whole cell equivalents were resolved by SDS-PAGE with 0.625 μg of recombinant Fbp-C and MBP-Fbp-N proteins used as controls for C1r and fibronectin, respectively. The SDS-PAGE resolved proteins were transferred to a PVDF membrane and then blocked overnight in 5% non-fat milk. The blots were washed and incubated with 20 μg of zymogen or active C1r for 1 hour. PVDF membranes were probed for C1r binding using a goat antibody to human C1r (R&D Systems) at a 1:3,000 dilution followed by a rabbit anti-goat antibody conjugated to HRP (Invitrogen) at a 1:5,000 dilution. Similar experimentation was done after incubation with 20 μg of human fibronectin with subsequent binding determined using a mouse monoclonal antibody specific for human fibronectin conjugated to HRP (Santa Cruz Biotechnology) at a 1:1,000 dilution. All blots were then visualized using the SuperSignal™ West Pico Plus chemiluminescent kit (Thermo Scientific). Control Western blots were performed as above with 2.5 x10^7^ whole cell equivalents to monitor Fbp expression and to serve as loading controls. To probe for FbpA and FbpB expression, mouse antibodies against FbpA or FbpB were used at a 1:5,000 dilution, respectively. To examine the flagellar protein FlaB, a mouse monoclonal antibody specific for FlaB (US Biologicals, Inc.) was used at a 1:20,000 dilution. Detection of membrane bound immune complexes was facilitated with a goat anti-mouse immunoglobulin HRP conjugate diluted 1:10,000 (Invitrogen). Visualization was the same as above. All Far Western blots were performed in duplicate.

### Crystallization, Structure Determination, Refinement, and Analysis

FbpA-C was concentrated to 12.8 mg/mL total protein in a buffer of (10 mM HEPES [pH 7.3], 50 mM NaCl). Crystals were obtained by vapor diffusion of sitting drops at 20°C by mixing 1 µL of protein with 1 µL of precipitant solution (0.1 M Tris [pH 8.5], 2 M ammonium sulfate). Large crystal plate clusters generally appeared after 9-10 d. Sequential rounds of micro-seeding produced samples of sufficient size and quality for harvesting. Samples were cryopreserved in precipitant solution supplemented with an additional 20% glycerol.

Crystallization trials using FbpB-C failed to produce diffraction quality crystals. Constructs removing residues from the N-terminus and C-terminus were screened resulting in identification of crystallization conditions for a construct of FbpB where five residues were removed from the C-terminus (*i.e.*, FbpB-Δ5C). FbpB-Δ5C was concentrated to 19.1 mg/mL and 8.69 mg/mL in 10 mM Tris (pH 8.0), 50 mM NaCl buffer. Crystals of FbpB-Δ5C were obtained by vapor diffusion of sitting drops at 4°C for both concentrations. Initial crystals grew in a condition containing 0.2M MgCl_2_, 0.1M Tris (pH 8.5), 30% PEG 4,000 and an optimized condition was identified by additive screening 4 mg/mL in 0.2 M MgCl_2_, 0.1 M Tris (pH 8.5), 26.25% PEG 4,000, 35 mM spermidine followed by microseeding. Crystals were harvested and cryoprotected by supplementing the crystallization buffer with 10% glycerol.

Monochromatic X-ray diffraction data were collected at 0.975Å (FbpA-C) or 1.00Å (FbpB-Δ5C) wavelength using beamline 22-ID of the Advanced Photon Source (Argonne National Laboratory). Diffraction data were integrated, scaled, and reduced using the HKL2000 software suite (53) and assessed for data quality (54) (**Table 3**). FbpA-C crystals grew in the space group P21 and contained two copies in the asymmetric unit which were related by twofold non-crystallographic symmetry. FbpB-Δ5C crystals grew in space group C2 and contained a single copy of FbpB-Δ5C in the asymmetric unit. Initial phases for FbpA-C were obtained by molecular replacement using a single copy of a poly-alanine model of BBK32-C (PBD:6N1L) generated with the program CHAINSAW *via* the CCP4 software package (55). Initial phases for FbpB-Δ5C were obtained by molecular replacement using a FbpA-C polyalanine model that was created using CHAINSAW from CCP4 (55). Following an initial round of refinement, models for FbpA-C and FbpB-Δ5C were completed by a combination of automated chain tracing using PHENIX.AUTOBUILD (56–58) and manual building into electron density maps using COOT (59). The final model was completed upon iterative cycles of manual rebuilding and refinement using PHENIX.REFINE (56–58). The N-terminal cloning artifact sequence for FbpA-C (Residues G-S-T-G-S) and FbpB-Δ5C, and residues 264-268, 355, 401-403, and 427 of FbpB-Δ5C were not modeled in the final refined structure due to poor electron density. Refined coordinates and structure factors have been deposited in the Protein Data Bank, Research Collaboratory for Structural Bioinformatics, Rutgers University (www.rcsb.org/) under PDB ID codes 7RPR for FbpA-C and 7RPS for FbpB-Δ5C. A description of crystal cell constants, diffraction data quality, and properties of the final models for FbpA-C and FbpB-Δ5C can be found in **Table 3**. Representations of the protein structures and electron density maps were generated by PyMol (www.pymol.org/).

**Table 3:** Refinement data for the solved FbpA and FbpB crystal structures.

Data collection and refinement		
Data collection	FbpA _(222–371)_	FbpB _(267-427)_
Space group	P 1 21 1	C 1 2 1
Cell dimensions		
a, b, c, Å	58.43, 40.32, 74.09	62.77, 46.21, 59.56
α, β, γ, ˚	90.00, 105.41, 90.00	90.00, 102.94, 90.00
Resolution, Å	50.0-1.90 (1.97-1.90)	50-2.09 (2.17-2.09)
*R_meas_*	0.166 (0.654)	0.115 (0.424)
*R*_pim_	0.068 (0.276)	0.054 (0.209)
*CC_1/2_*	0.988 (0.834)	0.995 (0.910)
I/*σ*I	13.2 (2.13)	20.2 (2.42)
Completeness, %	97.3 (97.7)	96.5 (93.5)
Redundancy	5.8 (5.3)	4.4 (3.5)
Refinement		
Resolution, Å	35.71-1.90	33.21-2.09
No. reflections	25,840	9,513
*R_work_/R_free_*	0.206, 0.232	0.225, 0.258
No. non-hydrogen atoms	2,694	1,288
Protein	2,492	1,252
Water	202	36
B-factors		
Protein	27.6	44.2
Water	33.5	45.5
Rmsd		
Bond lengths, Å	0.014	0.010
Bond angles, ˚	1.39	1.27

Values in parantheses refer to the highest-resolution shell.

### Circular Dichroism

Far-UV spectra were collected to assess the secondary structure for FbpA-C, FbpB-C, FbpA DA-C, and FbpB-Δ5C. Samples were diluted to 10 μM in 10 mM Na_3_PO_4_ based on previous protocols (60). Spectra for a buffer control were used to subtract background noise. Spectra were collected across a wavelength range of 180-300 nm, at 120 nm min^-1^, using 1 nm step, 0.5 sec response, and 1 nm bandwidth. Data was collected using a Chirascan V100 instrument with a square small volume quartz cuvette with a path length of 0.05 cm (Applied Photophysics, UK).

### Analytical Gel Filtration Chromatography

Analytical gel filtration was performed as before (34). 100 μg of FbpB-C and FbpB-Δ5C in a total volume of 500 μL were injected onto a Superdex 75 10/300 GL column (GE Healthcare) at a flowrate of 0.5 mL/min. The running buffer used was 10 mM HEPES (pH 7.3), 140 mM NaCl.

### C1r-CCP2-SP Enzyme Inhibition Assay

A colorimetric assay was used to monitor dose-dependent inhibition of C1r-CCP2-SP enzyme activity based off (33, 34). 1 μM Fbps-C were incubated with 15 nM C1r-CCP2-SP enzyme and 300 μM of the serine protease substrate Z-Gly-Arg-thiobenzyl (MP Biomedical) in HBS buffer (10 mM HEPES [pH 7.3] and 140 mM NaCl). Enzyme cleavage of Z-Gly-Arg-thiobenzyl was determined by incubating the reaction with 100 μM DTNB (5,5’-Dithiobis-[2-nitrobenzoic acid], Ellman’s reagent). The EnSight Multimode Plate Reader (PerkinElmer) was used to read the absorbance values for the reaction at 412 nm. Data were obtained in triplicate and normalized with 15 nM C1r-CCP2-SP lacking inhibitor as a positive control that represented the 100% amount of Z-Gly-Arg-thiobenzyl cleaved by C1r-CCP2-SP in 30 min at 25°C. Using a DTNB and Z-Gly-Arg-thiobenzyl only negative control allowed for subtraction of any non-specific colorimetric change.

### ELISA-Based Complement Inhibition Assay

An ELISA-based assay that utilizes IgM as a specific activator of the classical pathway as described (32–34, 50, 61), was used to determine the pathway’s downstream effect while in the presence of the Fbps. Data were obtained in triplicate and normalized using no inhibitor with 2% normal human serum (Innovative Research) as a positive control that represented the 100% amount of C4b deposition in 1 hour at 37°C. Non-specific interactions were subtracted out using a negative control with no serum. An EnSight Multimode Plate Reader (PerkinElmer) was used to read the absorbance values at 450 nm for the reaction.

### Hemolysis Assay

A hemolysis assay that uses antibody-opsonized sheep red blood cells to specifically activate the classical pathway was performed as previously described (32, 33, 62) to determine if *B. miyamotoi* Fbp-C proteins protected erythrocytes from complement-mediated hemolysis. A serial dilution series of Fbp-C proteins was incubated with buffer-exchanged pre-sensitized sheep erythrocytes (Complement Technology) and 2% normal human serum (Innovative Research). After a 1 hour incubation at 37°C the reaction was clarified *via* centrifugation at 500 x g and the absorbance read using an EnSight Multimode Plate Reader (PerkinElmer) at 412 nm. Data were obtained in triplicate normalized using a no inhibitor with serum positive control that represented the 100% cell lysis at 37°C in 1 hour. Background hemolysis was subtracted out using a negative control with only buffer and red blood cells.

### Quantitative ELISA to Assess *B. miyamotoi* Lipoproteins Binding to Serum C1r

The interaction of *B. miyamotoi* Fbp proteins with C1r in human serum was measured using an ELISA-type binding assay based on (63). Fbp proteins were immobilized overnight onto high-binding 96-well plates (Greiner Bio-One) at 0.2 mg/mL in 100 mM NaCO_3_ (pH 9.6). A twofold serial dilution of normal human serum (Innovative Research) ranging from 0.0012-10% serum in CP Assay buffer (10 mM HEPES [pH 7.3], 140 mM NaCl, 2 mM CaCl_2_, 0.5 mM MgCl_2_, 0.1% gelatin) was incubated with immobilized Fbp-C proteins at 37°C for 1 hour. The amount of serum C1r bound to immobilized Fbps was found using a goat antibody to human C1r (R&D Systems) diluted 1:2,000 and immune complexes detected with rabbit anti-goat Ig conjugated to HRP (Invitrogen) at a 1:3,000 dilution. Data were obtained in at least duplicate and read at 450 nm using the EnSight Multimode Plate Reader (PerkinElmer) with non-specific interactions subtracted out using a negative control with no serum.

### Proteinase K Accessibility Assays

*B. burgdorferi* strain B314 was grown to mid-log phase and harvested by centrifugation at 4,500 x g for 10 minutes at 4°C, and washed once with PBS. The cell pellet was resuspended in 0.8 mL of either PBS alone, or PBS with proteinase K to a final concentration of 200 μg/mL, and incubated at 20°C for 40 min. Reactions were terminated by the addition of phenylmethylsulfonyl fluoride (PMSF) to a final concentration of 1 mM. Cells were centrifuged (4,500 x g for 10 min at 4°C), washed twice with PBS, and resuspended in sample buffer. Samples corresponding to 5x10^7^ whole cell equivalents were run on SDS-PAGE gel, transferred to PVDF membranes and immunoblotted with mouse polyclonal antibodies to FbpA and FbpB or monoclonal antibodies directed against BBK32 or borrelial FlaB (US Biologicals Inc.) and imaged with chemiluminescence as previously described (33).

### Serum Complement Sensitivity Assay

Assays were performed as in (33, 34). Briefly, *B. burgdorferi* strain B314 producing BBK32 (pCD100), *B. miyamotoi* FbpA (pAP8) or FbpA-DA (pAP13), and *B. miyamotoi* FbpB (pAP11) (see **Table 1**), as well as the vector-only control B314 pBBE22*luc*, were grown at 32°C in 1% CO_2_ to early- to mid-log phase and diluted in complete BSK-II media to a final cell density of 1x10^6^ cells/mL. *B. burgdorferi* cells were then incubated in 15% normal human serum (Complement Technology) or heat inactivated serum (55°C for 30 min.), with thermal inactivation of complement proteins as a positive control for survival, for 1.5 hours at 37°C under gentle agitation. Cell survival was assessed by dark field microscopy based on cell motility and overt membrane damage or lysis.

### Soluble Protein Inhibition Rescue Assay

The ability of exogenous *B. miyamotoi* FbpA-C, FbpA DA-C, and FbpB-C to rescue serum-sensitive *B. burgdorferi* B314 pBBE22*luc* was measured similarly to the serum sensitivity assay. Exogenous complement inhibitory proteins were added at a final concentration of 48 nM, 240 nM, and 1.2 μM, approximately five-fold less than, equal to, and five-fold greater than the concentration of C1r in the human serum sample used. Survival was compared to that of B314 pBBE22*luc* with no exogenous protein addition, and of B314 pCD100, which produces BBK32, as described for the serum sensitivity assays.

### Statistics

Data is shown as the average of the replicates with 95% confidence intervals. Half-maximal inhibitory concentrations (IC_50_) values for the CP inhibitory ELISA and CP hemolysis assays were determined using a non-linear variable slope regression fit. The half-maximal effective concentration (EC_50_) for the serum C1r binding ELISA was determined using a non-linear one-site total regression fit. One-way ANOVA was used for the C1r enzyme and competition SPR experiments to determine differences among the means, followed by a *post-hoc* Tukey analysis for pairwise comparison of each Fbp protein. For serum sensitivity and B314 “rescue” assays, two-way ANOVA with a Šidák correction for multiple comparisons was used. All statistical analysis was performed using GraphPad Prism version 9.3.

## Results

### *B. miyamotoi* Encodes Two Orthologous Proteins to *B. burgdorferi* BBK32

**Figure 1:**
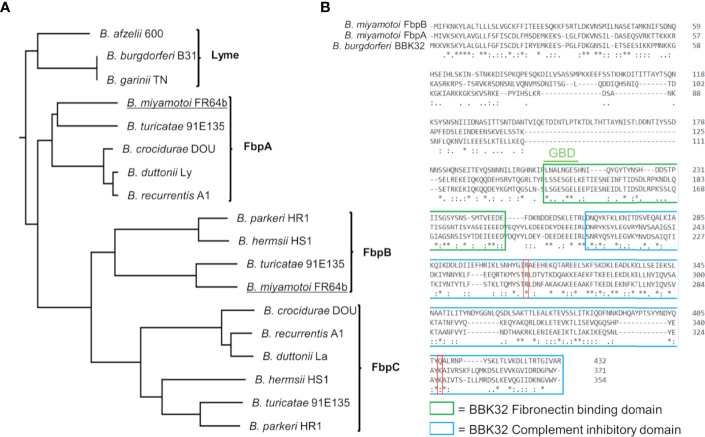
*B. miyamotoi* encodes two orthologous genes to *B. burgdorferi* BBK32. **(A)** BBK32 orthologs are found in relapsing fever (RF)-associated and *B. miyamotoi* spirochetes and are denoted FbpA, FbpB, and FbpC (41). *B. miyamotoi* FR64b FbpA and FbpB are underlined. **(B)** An alignment of *B. miyamotoi* strain FR64b FbpA and FbpB to *B. burgdorferi* strain B31 BBK32 shows differences of the amino acid sequences within the fibronectin binding (green box) and complement inhibitory domains (blue box). The gelatin-binding domain (GBD) of BBK32 is denoted. The key residues R248 and K327 of BBK32 involved in complement C1r binding are indicated by a red box. * conserved, : strongly similar, . weakly similar.

*B. burgdorferi* BBK32 interacts with multiple host proteins through non-overlapping protein domains. The N-terminus of BBK32 (BBK32-N) binds to fibronectin (35–37, 40, 64–70), while the C-terminal domain (BBK32-C) interacts with complement serine protease C1r (32, 33). Relapsing fever spirochetes encode a combination of up to three separate BBK32 orthologs termed fibronectin binding proteins (Fbps) (41) (**Figure 1A**). Fbp genes organize into three protein families denoted FbpA, FbpB, and FbpC and vary in their sequence identity to BBK32 between 56-62% (FbpAs), 25-30% (FbpBs), and 22-27% (FbpCs) (**Figure S1A**). A BLASTp search of the *B. miyamotoi* strain FR64b genomic sequence, using *B. burgdorferi* strain B31 BBK32 as a query, identified only two orthologous sequences; one belonging to the FbpA family and one to the FbpB family (**Figure 1A**). Unlike *B. miyamotoi* FbpA, FbpB lacks significant amino acid sequence homology for a key motif that is important for BBK32’s interaction with fibronectin (**Figure 1B**) (i.e., gelatin-binding domain sequence [GBD]) (69). Similarly, while a critical residue involved in the BBK32/C1r interaction is conserved in both FbpA and FbpB (i.e. BBK32-R248/FbpA-R264/FbpB-R309), another important ‘hot-spot’ lysine residue is conserved only in FbpA (BBK32-K327/FbpA-K343) (**Figure 1B**) (34).

### Differential Interactions of *B. miyamotoi* Fbps With Human Fibronectin

**Figure 2:**
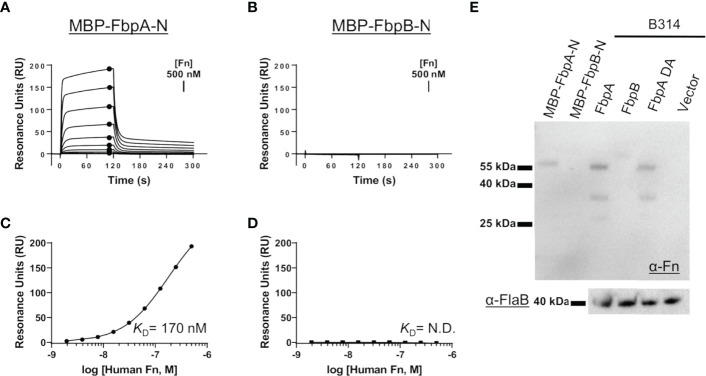
Assessing the interaction of human fibronectin with *B. miyamotoi* FbpA and FbpB. **(A, B)** SPR was used to quantitatively measure the interaction between FbpA-N **(A)** and FbpB-N **(B)** with human fibronectin. MBP-Fbp-N fusion proteins (Fbp sequences denoted by green box in **Figure 1B**) were immobilized on a sensorchip using amine coupling, then a twofold dilution series of purified human fibronectin ranging from 1.95-500 nM was injected. A representative sensorgram from the three replicates for each biosensor surface is shown. **(C, D)** Equilibrium dissociation constants (*K*_D_) were determined using steady state fits and are shown as the mean +/- standard deviation of three replicates in **Table 4** for FbpA **(C)** and FbpB **(D)**. Closed circles in **(A)** correspond to the steady-state response used for calculation of the steady state fits in **(C)**, respectively. A *K*_D_ value for the fibronectin interactions with MBP-FbpB-N was not determined (N.D.). **(E)** To assess if full-length Fbp proteins were capable of binding fibronectin, a Far Western approach was implemented by probing full-length Fbps from *B. burgdorferi* strain B314 “knock-in” lysates with fibronectin. Full-length FbpB fails to interact with fibronectin, whereas FbpA and FbpA DA do. A spirochete transformed with shuttle vector only (pBBE22*luc*) and recombinant MBP-FbpA-N/MBP-FbpB-N were used as controls. A Western blot against the flagellar protein FlaB (α-FlaB) was used as a loading control.

We hypothesized that the sequence variation in predicted functional sites of FbpB, and to a lesser degree FbpA, may result in altered function of *B. miyamotoi* Fbps relative to BBK32. To test this, we first began by evaluating the direct interaction of each protein with human fibronectin. We expressed and purified recombinant maltose-binding protein (MBP) fusion proteins that encoded the predicted fibronectin-binding site based on alignment to BBK32 (termed MBP-FbpA-N and MBP-FbpB-N) (**Figure 1B**, green box). Surface plasmon resonance (SPR) experiments were carried out by immobilizing each fusion protein on the surface of an SPR sensorchip and evaluating purified human fibronectin as an analyte in a dose-dependent manner. MBP-FbpA-N bound dose-dependently to soluble human fibronectin (*K*_D_ = 170 nM) (**Figures 2A** **, C**), whereas MBP-FbpB-N exhibited no detectable interaction with fibronectin at concentrations up to 500 nM (**Figures 2B** **, D**).

To exclude the possibility that FbpB may utilize a site outside of the predicted fibronectin-binding region to engage fibronectin, and to evaluate the interaction in the context of a full-length lipoprotein, we expressed intact *fbpA* and *fbpB* ectopically in *B. burgdorferi* strain B314. *B. burgdorferi* B314 is a serum-sensitive, high-passaged strain that has lost all linear plasmids including the 36 kilobase linear plasmid (lp36) that encodes *bbk32* (46). Equivalent protein lysates from *B. burgdorferi* strain B314 derivatives were tested for their ability to produce native *B. miyamotoi* FbpA, a double alanine mutant of FbpA at residues 264 and 343—R264A and K343A—denoted FbpA-DA (discussed below), and FbpB, by SDS-PAGE and Western immunoblot analysis (**Figures S2A, B**). Whereas the antibody to FbpA recognized only the FbpA protein, antisera to FbpB recognized both FbpB and, to a lesser extent, FbpA (**Figure S2A**). We then tested these same samples using a Far Western approach with human fibronectin as the probe. Both FbpA and FbpA-DA in B314 bound human fibronectin whereas samples containing FbpB or the pBBE22*luc* shuttle vector control spirochetes exhibited no detectable binding (**Figure 2E**). Consistent with the SPR experiments (**Figures 2A** **–D**), recombinant MBP-FbpA-N fusion protein bound human fibronectin in this assay format, whereas the MBP-FbpB-N protein did not (**Figure 2E**). Analysis of total lysate samples by SDS-PAGE (**Figure S2B**) and FlaB protein by Western blot (**Figure 2E**) demonstrated equivalent protein levels were used for comparison. These results show that while *B. miyamotoi* FbpA retains BBK32-like fibronectin-binding activity, FbpB does not.

### *B. miyamotoi* FbpA and FbpB Bind to Activated C1r in a BBK32-Like Manner

**Figure 3:**
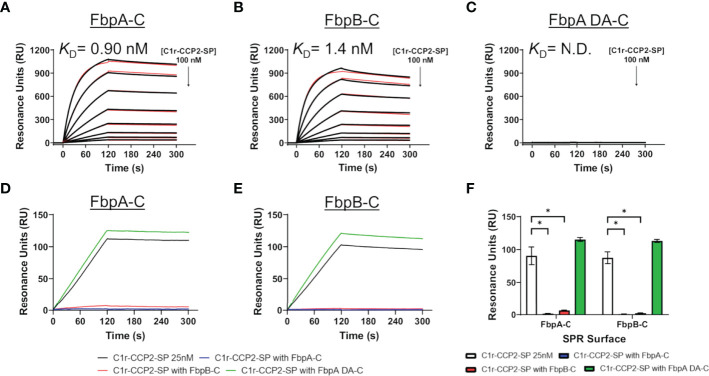
Assessing the interaction of human C1r with *B. miyamotoi* FbpA and FbpB. SPR was used to assess protein-protein interactions between the C-terminal regions of each Fbp protein and activated C1r-CCP2-SP. Immobilized **(A)** FbpA-C, **(B)** FbpB-C, and **(C)** FbpA-C-R264A-K343A (referred to as FbpA DA-C throughout) were subjected to an injection series of serially diluted C1r-CCP2-SP (0.78 - 100 nM). A representative sensorgram from the three replicates for each Fbp is shown with the black curve being the sensorgram and the red curve the associated kinetic fit. *K*_D_ values were determined using kinetic fits and are shown as the mean +/- standard deviation of three replicates in **Table 4**. A *K*_D_ value for C1r-CCP2-SP interactions with FbpA DA-C was not determined (N.D.). **(D, E)** Competition experiments were carried out using 25 nM C1r-CCP2-SP alone or mixed with 25 nM soluble Fbp proteins injected over FbpA-C **(D)** or FbpB-C **(E)**. **(F)** Comparisons of statistical significance were performed for data shown in panels **(D, E)** using a one-way ANOVA followed by a multiple comparison Tukey test (* = p < 0.05).

Next, we examined if *B. miyamotoi* FbpA and FbpB bound human C1r in a manner similar to *B. burgdorferi* BBK32 (32–34). BBK32 binds C1r *via* a C-terminal domain where residues R248 and K327 are critical in mediating the interaction with the S1 site of the C1r serine protease (SP) domain and the B-loop of C1r, respectively (34). Each of these key residues are conserved in FbpA (i.e., R264 and K343), whereas in FbpB, only the arginine is conserved (i.e., R309) (**Figure 1B**). We expressed and purified recombinant C-terminal truncation constructs of *B. miyamotoi* FbpA (FbpA-C), a site-directed double alanine mutant of FbpA-C (FbpA-R264A-K343A, denoted FbpA DA-C) (**Figure S2C**), and FbpB (FbpB-C), and assessed their direct interaction with a recombinant activated C1r construct encompassing the complement control protein 2 and serine protease domains (C1r-CCP2-SP). SPR binding assays showed that FbpA-C and FbpB-C bound with high affinity to C1r-CCP2-SP with *K*_D_ = 0.90 nM and 1.4 nM, respectively (**Figures 3A** **, B** and **Table 4**), whereas FbpA DA-C exhibited no detectable C1r-CCP2-SP binding (**Figure 3C**).

**Table 4:** SPR and complement assay results.

	MBP-FbpA-N	MBP-FbpB-N
**SPR (Affinity Fit): Human Fibronectin (*K*_D_)**	170 ± 5.4 nM	N.D.
	**FbpA-C**	**FbpB-C**
**SPR (Kinetic Fit):C1r-CCP2-SP (*K*_D_)**	0.90 ± 0.0017 nM	1.4 ± 0.024 nM
**SPR (Kinetic Fit):C1r-CCP2-SP (*k*_a_)**	3.3x10^5^ ± 3.6x10^3^ 1/Ms	3.9x10^5^ ± 5.3x10^2^ 1/Ms
**SPR (Kinetic Fit):C1r-CCP2-SP (*k*_d_)**	3.1x10^-4^ ± 2.0x10^-5^ 1/s	5.5x10^-4^ ± 8.8x10^-6^ 1/s
**Complement ELISA (IC_50_)**	23 (18 to 30) nM	1,900 (1,600 to 2,300) nM
**Erythrocyte Hemolysis (IC_50_)**	220 (170 to 280) nM	N.D.
**SPR (Kinetic Fit): C1r Zymogen (*K*_D_)**	0.85 ± 0.036 nM	N.D.
**SPR (Kinetic Fit): C1r Zymogen (*k*_a_)**	2.2x10^4^ ± 2.3x10^3^ 1/Ms	N.D.
**SPR (Kinetic Fit): C1r Zymogen (*k*_d_)**	1.9x10^-5^ ± 1.2x10^-6^ 1/s	N.D.
**SPR (Kinetic Fit): Active C1r (*K*_D_)**	0.39 ± 0.049 nM	1.1 ± 0.042 nM
**SPR (Kinetic Fit): Active C1r (*k*_a_)**	6.8x10^4^ ± 1.0x10^3^ 1/Ms	6.8x10^4^ ± 8.0x10^2^ 1/Ms
**SPR (Kinetic Fit): Active C1r (*k*_d_)**	2.7x10^-5^ ± 2.3x10^-6^ 1/s	7.6x10^-5^ ± 3.6x10^-6^ 1/s
**Serum C1r Binding (EC_50_)**	0.27 (0.23 to 0.32) %	N.D.

95% Confidence interval = (), Standard deviation (S.D.)= ± S.D., N. D., Not Determined.

Next, we determined if FbpA-C and FbpB-C could compete with one another for binding to C1r-CCP2-SP. Equimolar mixtures of C1r-CCP2-SP with soluble FbpA-C, FbpA DA-C or FbpB-C were injected over immobilized FbpA-C and FbpB-C. Significantly reduced binding signals were observed when soluble FbpA-C or FbpB-C was used, but not for FbpA DA-C, indicating that active FbpA and FbpB proteins are capable of competing with one another for binding to activated C1r (**Figures 3D–F**). These results suggest that both FbpA and FbpB bind to activated C1r in a manner similar to what has been reported for BBK32 and underscores the importance of the conserved R248 and K327 residues (BBK32 numbering) in the FbpA/C1r interaction (34). Interestingly, although BBK32-K327 is not conserved in FbpB (**Figure 1B**), this did not preclude high-affinity interaction with activated C1r-CCP2-SP.

### Differential Inhibition of Human Classical Pathway Complement Activation by *B. miyamotoi* FbpA and FbpB

**Figure 4:**
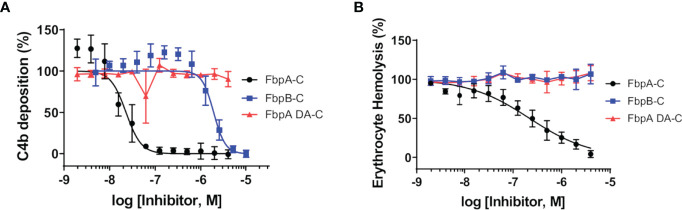
Assessing the complement inhibitory properties of *B. miyamotoi* FbpA and FbpB *in vitro*. **(A)** An ELISA-based assay specific for CP complement activation was used to determine if FbpA-C and FbpB-C were able to inhibit human CP progression. FbpA-C, FbpA DA-C, and FbpB-C, were serially diluted two-fold then incubated with normal human serum, followed by detection of deposited C4b. IC_50_ values were determined using normalized non-linear regression analysis and reported in **Table 4** along with 95% confidence intervals. **(B)** A CP hemolysis assay was carried out using a two-fold serial dilution of Fbp proteins incubated with pre-sensitized sheep erythrocytes and 2% normal human serum. MAC formation and progression of the CP was measured by detecting MAC-mediated erythrocyte hemolysis. IC_50_ values were determined using normalized non-linear regression analysis and reported in **Table 4** along with 95% confidence intervals.

*B. miyamotoi* FbpA-C and FbpB-C were next assessed for the ability to inhibit the classical complement cascade due ostensibly to the inhibition of C1r. A classical pathway-specific ELISA-based assay was implemented with Fbp-based inhibitors and normal human serum (32–34). As expected, in light of the C1r-CCP2-SP binding data (**Figure 3**), FbpA-C demonstrated potent inhibition of classical pathway-mediated complement activation (IC_50_ = 23 nM), whereas the FbpA DA-C mutant lost all inhibitory activity (**Figure 4A** and **Table 4**). FbpB-C blocked complement activation but, surprisingly, was ~80-fold less potent than FbpA (IC_50_ = 1,900 nM) despite having similar high affinity for activated C1r-CCP2-SP. To confirm these activities, each protein was serially diluted and incubated with opsonized sheep erythrocytes and normal human serum. Consistent with the ELISA-based assay, FbpA, but not FbpA DA prevented hemolysis (**Figure 4B**). In this assay format, FbpB-C had even lower activity, showing no inhibition at concentrations up to 4µM.

### Structural Differences Between *B. miyamotoi* FbpA-C and FbpB-C

**Figure 5:**
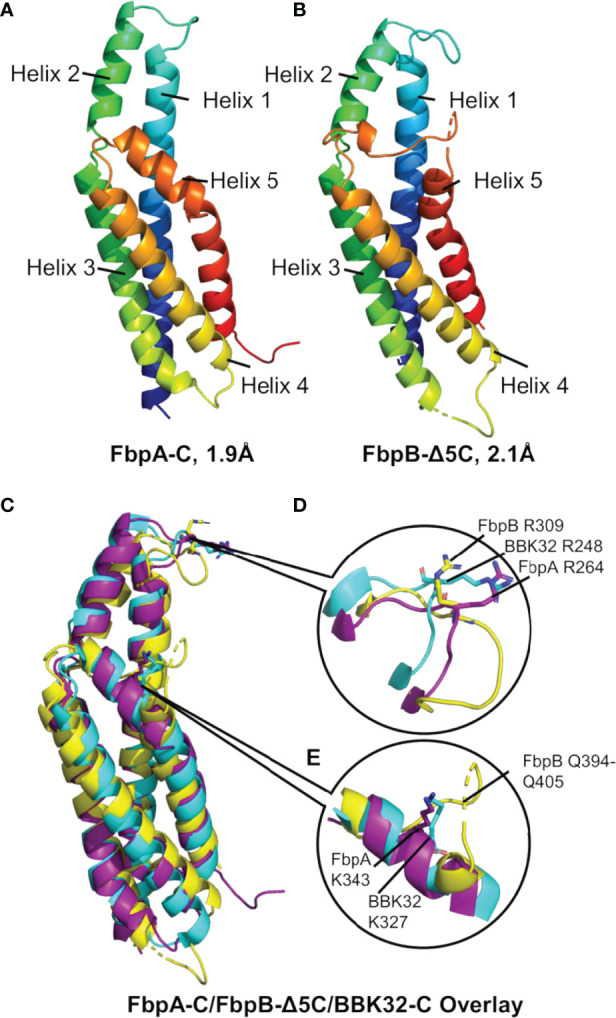
The crystal structures of *B. miyamotoi* FbpA-C and FbpB-Δ5C. Crystal structures of **(A)** FbpA-C (PDB Code: 7RPR) and **(B)** FbpB-Δ5C (PDB Code: 7RPS) were solved to a limiting resolution of 1.9Å and 2.1Å, respectively. Both FbpA-C and FbpB-Δ5C are shown as a color spectrum where blue represents the N-terminus and red represents the C-terminus. **(C)** A structural alignment of FbpA-C (purple), FbpB-Δ5C (yellow), and *B*) *burgdorferi* BBK32-C (cyan, PDB Code: 6N1L). **(D)** Each protein is structurally similar near the functionally critical BBK32-R248 residue (i.e., FbpA-R264 and FbpB-R309). **(E)** Differences between BBK32-C, FbpA-C, and FbpB-C are found at the functionally critical BBK32-K327 residue (K343 in *B miyamotoi* FbpA). FbpB-Δ5C shows an altered secondary structure with an extended loop structure corresponding to residues Q394-Q405 that aligns to the alpha helical structures found in both FbpA-C and BBK32-C.

While differences in the ability of *B. miyamotoi* FbpA and FbpB to bind fibronectin are likely attributed to low sequence conservation of critical fibronectin-binding motifs in FbpB (**Figure 1B**) (36, 37, 66, 69, 70), the basis for differences in the complement inhibitory activities of FbpA-C and FbpB-C were less clear. To determine if FbpA and FbpB have altered structures in their C1r-binding C-terminal regions, we solved the crystal structures of each protein. FbpA-C crystals diffracted to a limiting resolution of 1.9Å (R_free (%)_/R_work (%)_ = 20.6/23.2) (PDB: 7RPR) (**Figure 5A**, **Figures S3A, B** and **Table 3**). FbpB-C was recalcitrant to crystallization; however, an active FbpB-C construct lacking five C-terminal residues (**Figure S4**, referred to as FbpB-Δ5C hereafter), was crystallized and the structure resolved to a limiting resolution of 2.1Å (R_free (%)_/R_work (%)_ = 22.5/25.8) (PDB: 7RPS) (**Figure 5B**, **Figures S3C, D** and **Table 3**).

A structural overlay with BBK32-C (PDB: 6N1L) and *B. miyamotoi* FbpA-C and FbpB-Δ5C reveals that all three proteins have similar folds involving a core four-helix bundle (helices 1, 3, 4, and 5) with alpha helix 2 extending away from the core (**Figure 5C**). BBK32-C was previously shown to interact with activated C1r using two primary binding sites: i) the active site of C1r involving several BBK32 residues, including R248, and ii) the B-loop of C1r involving residues on the fifth alpha helix, including K327 (34). A structural overlay of FbpA-C, FbpB-Δ5C, and BBK32-C shows that the three proteins retain an overall similar structure around the conserved R248 residue (BBK32 numbering) (**Figure 5D**). However, we noted that the area near BBK32-K327 involves a minor structural alteration in FbpA-C and a more major alteration in FbpB-Δ5C (**Figure 5E**). In FbpA-C, the kinked portion of alpha helix 5 extends one turn longer than in BBK32, resulting in FbpA-C containing an additional surface-exposed residue on this helix (**Figure 5E**). More strikingly, the structure of FbpB-Δ5C has a significantly altered secondary structure in this region when compared to both FbpA-C and BBK32-C (**Figure 5E**). Specifically, FbpB-Δ5C shows a loss of secondary structure within alpha helix 5 that accommodates a loop insertion sequence (i.e., residues Q394-Q405), which are not conserved in both BBK32-C or FbpA-C. Furthermore, a proline residue (P397) in this sequence interrupts the alpha helical secondary structure in FbpB-Δ5C. A portion of this unique loop structure in FbpB has apparent increased disorder in the crystal structure, indicated by weak electron density surrounding residues 401-403 in this region of the protein. Collectively, these data show that FbpA and FbpB are structurally divergent from BBK32 at a functionally critical region of the protein on the fifth alpha helix (**Figures 5C** **–E**).

### *B. miyamotoi* FbpA and FbpB Differentially Recognize C1r Activation States

C1r is a serine protease subcomponent of the C1 complex which circulates in blood as an inactive zymogen, and is converted to an activated enzyme by autocatalysis when C1 binds to an activating ligand (71–75). This transition involves a conformational change in the SP domain of C1r resulting in a rearrangement of several loop structures, including the C1r B-loop (75) (**Figure S5**). We have previously shown that BBK32 binds to both active and zymogen forms of C1r in a manner that relies on R248 and K327 (34). Given the involvement of B-loop residues in the BBK32-K327 binding site, the observed structural differences at this site in the Fbp crystal structures (**Figure 5**), and the potential for different conformations of this loop between zymogen and active states of C1r (**Figure S5**) (75), we hypothesized that FbpA and FbpB may have altered recognition properties for zymogen C1r.

**Figure 6:**
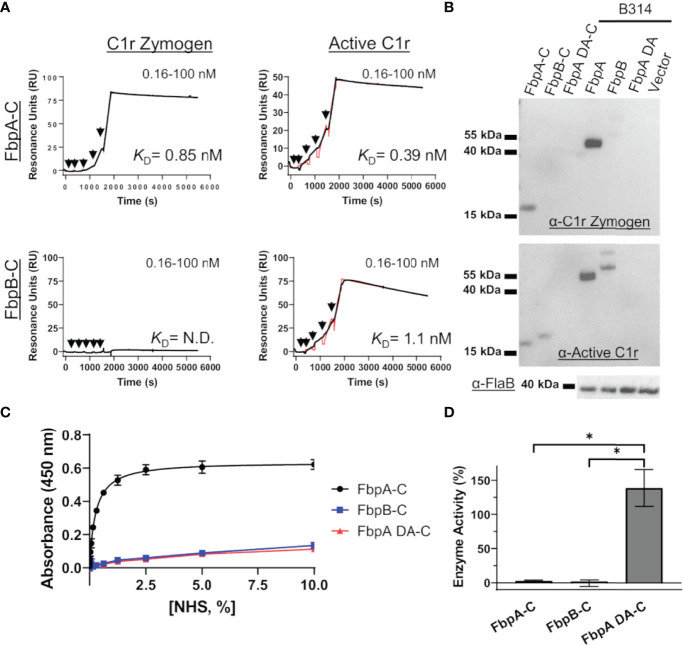
FbpA-C and FbpB-C interact differentially with zymogen and activated forms of human C1r. **(A)** Single cycle SPR was used to determine binding affinities of C1r zymogen or active C1r ranging from 0.16 - 100 nM injected over immobilized Fbps. A representative sensorgram from a three-injection series for each Fbp-C and C1r active state is shown with the black curve being the sensorgram and the red curve the associated kinetic fit. *K*_D_’s were determined using kinetic fits and are shown as the average and standard deviation of three replicates in **Table 4**. **(B)** Far Western overlays using *B. burgdorferi* B314 lysates probed with zymogen or enzymatic forms of purified full-length C1r, followed by an antibody to C1r. **(C)** An ELISA-type binding assay was used to determine the ability of FbpA-C, FbpA DA-C and FbpB-C to interact with C1r in human serum. FbpA-C (black), FbpB-C (blue), and FbpA DA-C (red) were immobilized on an ELISA plate then incubated with a two-fold dilution series of normal human serum ranging from 0.0012-10%. Corresponding EC_50_ values for FbpA-C, FbpB-C, and FbpA DA-C are reported in **Table 4**. **(D)** Fbp-mediated inhibition of purified active C1r-CCP2-SP enzymatic activity was assessed by incubating 15 nM C1r-CCP2-SP with a 1 μM of FbpA-C (white), FbpB-C (striped), and FbpA DA-C (grey). C1r-CCP2-SP enzymatic activity was determined by incubation with a C1r substrate (Z-Gly-Arg-sBzl) that, once cleaved, reacts with DTNB resulting in a colorimetric change. No statistical difference was found between FbpA-C and FbpB-C inhibitory activity, whereas FbpA DA-C exhibited significant loss in inhibitory activity. Statistical analysis was performed using a one-way ANOVA followed by a multiple comparison Tukey test (*=p<0.05).

To assess this, *B. miyamotoi* FbpA-C and FbpB-C were immobilized on an SPR chip and a five-fold dilution series of either zymogen or active forms of full-length purified C1r were injected over each surface. Interestingly, while FbpA-C was able to bind to both zymogen and active forms of C1r readily (C1r zymogen *K*_D_ = 0.85 ± 0.036 nM, active C1r *K*_D_ = 0.39 ± 0.049 nM), FbpB selectively interacted with active C1r only (active C1r *K*_D_= 1.1 ± 0.042 nM) (**Figure 6A**). We next confirmed that the selective nature of FbpB was observed with full-length proteins expressed in *B. burgdorferi* B314 strains in an approach identical to that presented for fibronectin in **Figure 2E**. Whereas FbpA could recognize both zymogen and activated forms of purified C1r, FbpB preferentially bound activated C1r. The negative controls, FbpA DA and vector-only lysates, did not bind either form of C1r as expected (**Figure 6B**), although we note that FbpA DA retained fibronectin binding (**Figure 2E**).

We considered whether the apparent zymogen/active C1r selectivity differences between FbpA and FbpB could be observed in serum where C1r is in a zymogen form (71–74). To test this, we implemented a serum-based ELISA-type binding assay. *B. miyamotoi* FbpA-C, FbpB-C, and FbpA DA-C were immobilized onto an ELISA plate and incubated with a dilution series of normal human serum followed by detection of C1r binding using a C1r antibody. FbpA-C readily bound serum C1r (half-maximal effective concentration (EC_50_) = 0.27% (v/v) serum concentration), while FbpB-C and FbpA DA-C did not (**Figure 6C**). However, consistent with the ability of FbpB-C to bind tightly to previously activated forms of C1r (**Figures 3**, **6A**), FbpB-C inhibited the enzymatic activity of pre-activated C1r-CCP2-SP enzyme similar to FbpA-C (**Figure 6D**). Collectively, these results show that, like BBK32, *B. miyamotoi* FbpA can bind and inhibit both the zymogen and active forms of C1r, whereas *B. miyamotoi* FbpB selectively interacts with and inhibits only the active form of the C1r protease.

### *B. miyamotoi* Fbp Proteins Protect a Serum-Sensitive Strain of *B. burgdorferi* From Complement-Mediated Killing

**Figure 7:**
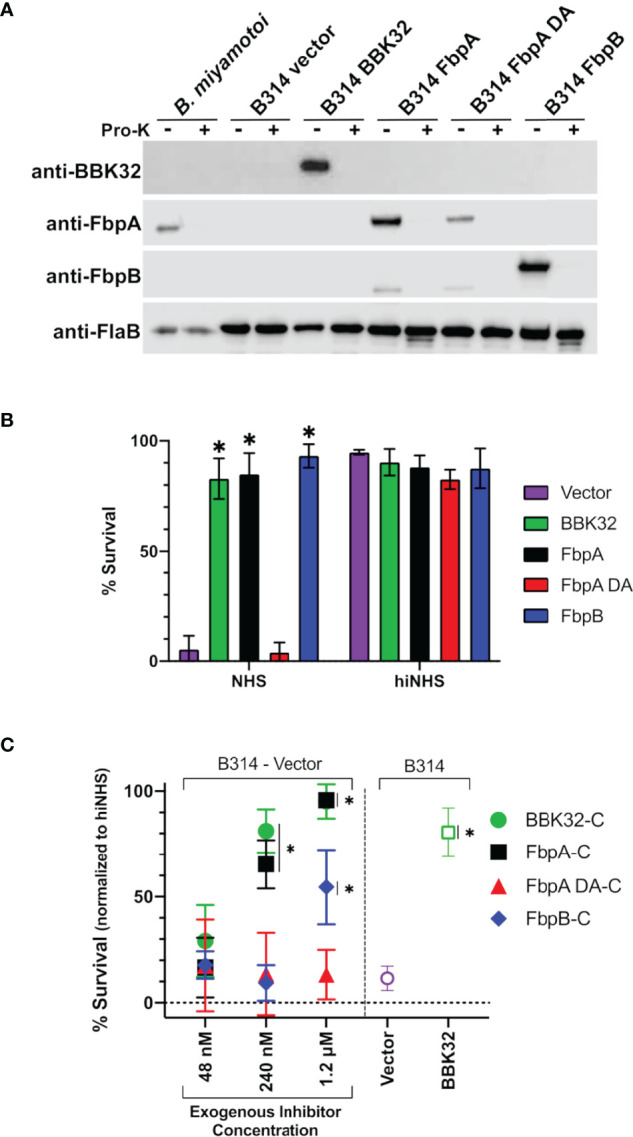
Determining the ability of surface-expressed *B. miyamotoi* FbpA and FbpB to protect a serum-sensitive strain of *B. burgdorferi*. **(A)** Western blots of lysates from *B. miyamotoi* strain FR64b and *B burgdorferi* B314 isolates expressing *fbpA*, *fbpA-DA*, *fbpB*, and *bbk32* were probed with antibodies to FbpA (anti-FbpA), FbpB (anti-FbpB), BBK32 (anti-BBK32) and FlaB (anti-FlaB), the latter as a loading control and a control for a subsurface target. Vector refers to the plasmid-only backbone sample (B314/pBBE22*luc*). Samples from each population were subjected to proteinase K accessibility treatments to determine the surface expression of the proteins. Due to its periplasmic location, the flagellar protein, FlaB, is unaffected by proteinase K in intact cells and depicts structural integrity in the treated *B. miyamotoi* and B314 derivatives listed. **(B)** *B. miyamotoi* Fbp proteins were tested for their ability to confer resistance to normal human serum (NHS) sensitive *B. burgdorferi* strain B314 relative to vector-only and BBK32-expressing controls (negative and positive controls, respectively). Asterisks depict a significant increase in survival relative to FbpA DA and the vector control (*=p<0.0001). All strains exposed to heat inactivated NHS (hiNHS), rendering complement proteins inactive, were largely unaffected. **(C)** Rescue assays were used to determine if soluble exogenous recombinant proteins could promote survival of serum-sensitive vector-containing *B. burgdorferi* B314. Increasing five-fold concentrations of BBK32-C, FbpA-C, FbpA DA-C, and FbpB-C were added in serum sensitivity assays. The middle samples (i.e., 240 nM) have added recombinant protein that is roughly equivalent to the concentration of C1r in the assay conditions employed. Cells were assessed *via* darkfield microscopy. Included as controls are a vector-only strain of B314 with no exogenous protein addition, as well as a BBK32-producing B314 isolate (right side). Asterisks depict a significant increase in survival relative to the vector control (*=p<0.015). Significance for serum sensitivity and rescue assays was determined using two-way ANOVA with a Šidák correction for multiple comparisons.

Next, we assessed the protection *B. miyamotoi* FbpA and FbpB conferred to *B. burgdorferi* B314 when ectopically expressed on the cell surface. Since *B. miyamotoi* has not yet been genetically manipulated, we modeled these proteins’ surface expression and activity in B314 relative to a pBBE22*luc* vector-only control and cells producing BBK32, given that BBK32 confers serum resistance to strain B314 (32–34). As a prelude to the serum sensitivity assay, we first tested whether *B. miyamotoi* FbpA, FbpA DA or FbpB were surface exposed in *B. burgdorferi* strain B314 relative to a B314 BBK32 control, as well as expressed on the surface of *B. miyamotoi* strain FR64b, using the well-established proteinase K accessibility assay (32, 76). The results clearly show that FbpA, FbpA DA, FbpB, and BBK32 were all produced and are all protease-sensitive, indicating surface exposure in strain B314. However, only FbpA is detected on the surface of *B. miyamotoi* strain FR64b, potentially due to the *in vitro* growth conditions not being conducive for expression of *fbpB* (**Figure 7A**). Importantly, the subsurface endoflagellar protein, FlaB, was unaffected by the treatment in both *B. miyamotoi* strain FR64b and *B. burgdorferi* B314, indicating the preserved structural integrity of the borrelial cells tested (**Figure 7A**). As observed in **Figure S2A**, antibody to *B. miyamotoi* FbpB also recognized FbpA and FbpA DA, albeit to a lesser extent (**Figure 7A**).

We then exposed each B314 knock-in strain to normal human serum and assessed the viability of the cells. *B. miyamotoi* FbpA conferred levels of resistance comparable to BBK32, while the FbpA DA strain was as sensitive to serum killing as the vector-only control (**Figure 7B**). In contrast to the lower apparent inhibitory activity of recombinant FbpB-C (**Figure 4**), *B. burgdorferi* strain B314 producing FbpB on the surface also conferred significant protection against human serum (**Figure 7B**). In order to more fully address differences in the resistance observed for FbpB in the prior assays, we incubated serum sensitive B314 pBBE22*luc* with differential levels of *B. miyamotoi* FbpA-C, FbpB-C, FbpA DA-C, and *B. burgdorferi* BBK32-C to ask whether these soluble components could provide resistance to complement-dependent killing with normal human serum and thus “rescue” this sensitive *B. burgdorferi* strain. Exogenous addition of the C-terminus of the *B. miyamotoi* Fbps and *B. burgdorferi* BBK32-C resulted in dose-dependent rescue for BBK32-C, FbpA-C, and FbpB-C (**Figure 7C**). However, the inhibitory effect was weaker for FbpB-C, with significant inhibition observed only at the 1.2 µM treatment, which is comparable to the IC_50_ value observed for FbpB-C in the ELISA-based inhibition assay (**Figure 4**). Consistent with its inability to inhibit complement, addition of FbpA DA-C was unable to rescue serum-based killing of B314 pBBE22*luc* cells at any of the protein concentrations used (**Figure 7C**). Collectively, these data demonstrate that ectopically-produced *B. miyamotoi* FbpA and FbpB confer resistance to human serum.

## Discussion

Like other tick-borne microbial agents, *B. miyamotoi* spirochetes must survive host immune defenses, including complement, beginning at the tick bloodmeal stage and throughout host infection. *B. miyamotoi* has been reported to evade the alternative pathway (AP) of complement through recruitment of endogenous complement regulators or plasmin, and produces lipoproteins that directly bind to and inhibit complement proteins, like CbiA, to protect the spirochete from complement-mediated killing (21, 22, 77). However, little is known about how *B. miyamotoi* defends against the classical pathway (CP), which may be particularly important during later stages of infection when an adaptive immune response has been developed. Interestingly, the Lyme disease spirochete *B. burgdorferi* has evolved at least two mechanistically distinct inhibitors of the classical complement pathway, i) BBK32 (32–34) and ii) ElpB/ElpQ (78). As *elp* genes with CP inhibitory activity have yet to be identified in *B. miyamotoi* genomes, we hypothesized that *B. miyamotoi* may use orthologs of BBK32 to neutralize the classical complement cascade and/or exert BBK32-like fibronectin-binding activity (32–34).

*B. miyamotoi* strain FR64b encodes two BBK32 orthologs, FbpA and FbpB, that share 56% and 25% amino acid sequence identity to *B. burgdorferi* strain B31 BBK32, respectively (**Figures 1B**, **S1**). *B. miyamotoi* strain LB-2001, a North American strain of *B. miyamotoi* (24), also encodes FbpA and FbpB proteins with 85% and 81% identity to FR64b FbpA and FbpB, respectively. We found that *B. miyamotoi* strain FR64b FbpA exhibits similar activity to BBK32 for both fibronectin-binding and C1r-binding/inhibition. The results presented herein highlight that FbpA and BBK32 are multifunctional proteins capable of interacting with more than one host protein *via* separate domains (32–37, 40, 64–68). Similar to what has been proposed for *B. burgdorferi* BBK32, the anti-C1r activity found here for FbpA may selectively protect *B. miyamotoi* from CP-mediated complement killing. This is supported by the ability of FbpA to protect previously serum-sensitive spirochetes from complement-mediated bacteriolysis (**Figure 7B**). The functional consequence of the fibronectin-binding activity of *B. miyamotoi* FbpA interacting with fibronectin may be related to extravasation as has been shown for BBK32 (35–37, 40, 64–68), and/or it may serve other functions including acting as an adhesin to the vascular endothelium or binding to plasma fibronectin, another activity reported for BBK32 (38, 65, 79).

The functional consequence of *B. miyamotoi* FbpA and FbpB production within a host will need to be investigated *in vivo*. A current limitation is the lack of an immunocompetent mouse model for *B. miyamotoi* to determine the temporal expression of *fbpA* and *fbpB* during mammalian infection. The culturing of *B. miyamotoi* is possible (24), but challenging, and allows for only limited experimentation. Additionally, genetic manipulation of *B. miyamotoi* has not yet been reported. To overcome these challenges, we used an established human serum sensitivity assay in which serum resistance can be quantitatively measured and correlated with protein production of *B. miyamotoi* FbpA and FbpB on the surface of the serum-sensitive *B. burgdorferi* strain B314, e.g., as a “knock-in” construct (**Figure 7B**) (32–34). Alternatively, we tested whether the addition of exogenous FbpA-C, FbpA DA-C, or FbpB-C could “rescue” normal human serum-dependent killing of a sensitive B314 derivative (**Figure 7C**). The “knock-in” system is a minimalist approach that measures the complement inhibitory activity of surface-exposed proteins due to the absence of all linear plasmid-encoded lipoproteins and the concomitant absence of redundant immune evasion mechanisms. Proteinase K accessibility assays confirmed the surface localization of *B. miyamotoi* FbpA, FbpA DA, and FbpB in the surrogate *B. burgdorferi* B314 knock-in background (**Figure 7A**). Interestingly, only FbpA was produced following *in vitro* cultivation of *B. miyamotoi* strain FR64b and surface-expression was also confirmed; no FbpB was detectable by Western immunoblotting (**Figure 7A**). Limited cultivation conditions were tested here for *B. miyamotoi* and additional experimentation using mammalian-like growth conditions may also induce FbpB production comparable to that observed for pathogenesis-associated proteins in *B. burgdorferi* (80).

In contrast to *B. miyamotoi* FbpA, we found that FbpB fails to interact with human fibronectin in a BBK32-like manner and lacks a key motif that is known to be important for BBK32 recognition of fibronectin (37, 66) (**Figure 2**). The C1r-binding activity for FbpB proved to be more complex. Unlike BBK32/FbpA, FbpB fails to interact with zymogen forms of human C1r but instead selectively binds and inhibits the activated form (**Figures 3**, **6A, B, D**). The crystal structures presented here of FbpA-C and FbpB-C show that a defining feature of the C1r-binding domains for BBK32 and *B. miyamotoi* Fbps is a four-helix core with an extended second alpha helix that presents a functional arginine residue on a small surface-loop (**Figures 5C** **, D**). However, the crystal structure of FbpB revealed structural differences in the fifth alpha helix relative to FbpA and BBK32. FbpB has a divergent loop structure in this region and therefore lacks the same secondary structure seen in both FbpA and BBK32 (**Figure 5E**). We have previously shown that residues on the fifth alpha helix of BBK32 interact with a non-active site loop on C1r known as the B-loop (34). Despite these structural differences, FbpB-C was still capable of saturable inhibition of human complement activation, although at several orders of magnitude weaker potency in the ELISA-based assay (**Figure 4A**). Recombinant FbpB-C also protected serum sensitive spirochetes from complement-mediated killing, but was significantly weaker in its ability to do so relative to FbpA-C (**Figure 7C**). Initially, these results pointed to FbpA being the more relevant classical pathway complement inhibitor in *B. miyamotoi* and suggested that targeting of the zymogen form of human C1r is an important functional feature of this class of inhibitors. However, in our serum sensitivity assays, where *B. miyamotoi* FbpA and FbpB were produced ectopically on the surface of *B. burgdorferi*, both proteins conferred significantly greater protection to B314 than FbpA DA or the vector-only control (**Figure 7B**). These data suggest that within the physiological context of complement activation on the spirochete surface, FbpB’s inhibition of activated C1r is sufficient to prevent downstream complement activation. Thus, the selective recognition of activated C1r by FbpB implies that it functions at a different temporal or spatial level than FbpA. Combined with the differences in relative fibronectin-binding activities of FbpA and FbpB, our data suggest that Fbp proteins have evolved to have partially overlapping, but non-identical functions compared to the Lyme disease-associated BBK32 orthologs.

Nonetheless, *B. miyamotoi* FbpB’s conferred resistance in the human serum sensitivity assays was initially surprising based on *in vitro* biochemical data showing very limited inhibition of serum-based complement activation (**Figure 4B**). The lack of consensus between FbpB activity in the human serum sensitivity assay relative to FbpB-C likely reflects differences in the truncated soluble form of the recombinant protein relative to the full-length lipoprotein-tethered surface-exposed configuration (**Figure 7B**). Given that soluble FbpB-C significantly protected serum-sensitive spirochetes at high concentrations (**Figure 7C**), one explanation may be that differences observed in the B314 background are related to sufficient protein concentrations of surface-exposed FbpB that can confer complement resistance to these borrelial cells (**Figure 7B**). Another possibility is suggested by the observation that soluble FbpB-C does not interact with zymogen C1r in serum (**Figure 6C**), but rather interacts and inhibits C1r only once activated (**Figure 6**). Thus soluble forms of FbpA, but not FbpB, are capable of binding to C1r prior to surface activation. This difference likely makes the inhibitory activity of FbpB more dependent on surface localization compared to FbpA, which binds tightly to zymogen C1r in solution (**Figure 6C**).

In summary, we have characterized the structure and function of two *bbk32* orthologs within the emerging pathogen *B. miyamotoi*, termed FbpA and FbpB. Our investigation showed that FbpA and FbpB share some activities with one another and with *B. burgdorferi* BBK32. However, important differences – both structurally and at the functional level – were found to influence their relative ability to interact with both human fibronectin and complement C1r. Surprisingly, our data suggest that *B. burgdorferi* BBK32 and *B. miyamotoi* Fbp proteins have evolved distinct molecular mechanisms across the family of inhibitors. These observations have significance for understanding how *B. miyamotoi* modulates the host immune response and provides novel insight into the diverse structure-function relationships of this multifunctional class of borrelial lipoproteins.

## Data Availability Statement

The datasets presented in this study can be found in online repositories. The names of the repository/repositories and accession number(s) can be found below: http://www.wwpdb.org/, 7RPR, http://www.wwpdb.org/, 7RPS.

## Ethics Statement

The animal work described herein was reviewed and approved by Texas A&M IACUC.

## Author Contributions

CB and AP-P are equal contributors. JS and BG are co-corresponding authors. JS and BG designed research. CB and AP-P conducted research. AP-P, JS, BG CB analyzed data. CB, AP-P, JS, and BG wrote the paper.

## Funding

Support was provided by Public Health Service Grant R01-AI146930 from the National Institute of Allergy and Infectious Diseases (to JS and BG). X-ray diffraction data were collected at Southeast Regional Collaborative Access Team 22-ID beamline at the Advanced Photon Source, Argonne National Laboratory. Supporting institutions may be found at www.ser-cat.org/members.html.

## Conflict of Interest

The authors declare that the research was conducted in the absence of any commercial or financial relationships that could be construed as a potential conflict of interest.

## Publisher’s Note

All claims expressed in this article are solely those of the authors and do not necessarily represent those of their affiliated organizations, or those of the publisher, the editors and the reviewers. Any product that may be evaluated in this article, or claim that may be made by its manufacturer, is not guaranteed or endorsed by the publisher.
