# Supplementary material for: Borrelia miyamotoi FbpA and FbpB Are Immunomodulatory Outer Surface Lipoproteins With Distinct Structures and Functions
Source: Front Immunol. 2022 May 27;13:886733. doi: 10.3389/fimmu.2022.886733 (PMC9186069; doi:10.3389/fimmu.2022.886733)
Supplement: Supplementary file 1 [file DataSheet_1.pdf]

A

	FbpA	FbpB	BBK32
FbpA		27	70
FbpB	46		42
BBK32	56	25	

%Similarity

% Identity

B

	FbpA-N	FbpB-N	BBK32-N
FbpA-N		44	64
FbpB-N	25		37
BBK32-N	53	24	

%Similarity

% Identity

C

	FbpA-C	FbpB-C	BBK32-C
FbpA-C		50	76
FbpB-C	30		48
BBK32-C	59	26	

%Similarity

% Identity

Supplemental Figure 1. *B. miyamotoi* strain FR64b FbpA, FbpB and *B. burgdorferi* strain B31 BBK32 percent identity and similarity. Multiple sequence alignments were performed for (A) Full-length, (B) the N-terminus, and (C) the C-terminus of *B. miyamotoi* FR64b FbpA and FbpB, and *B. burgdorferi* B31 BBK32. The N-terminus was all sequence upstream of the C-terminus within the blue box in **Fig. 1B**, and the C-terminus was the sequence corresponding to the blue box in **Fig. 1B**. Alignments were generated using Clustal Omega from EMBL-EBI with percent identity and percent similarity shown.

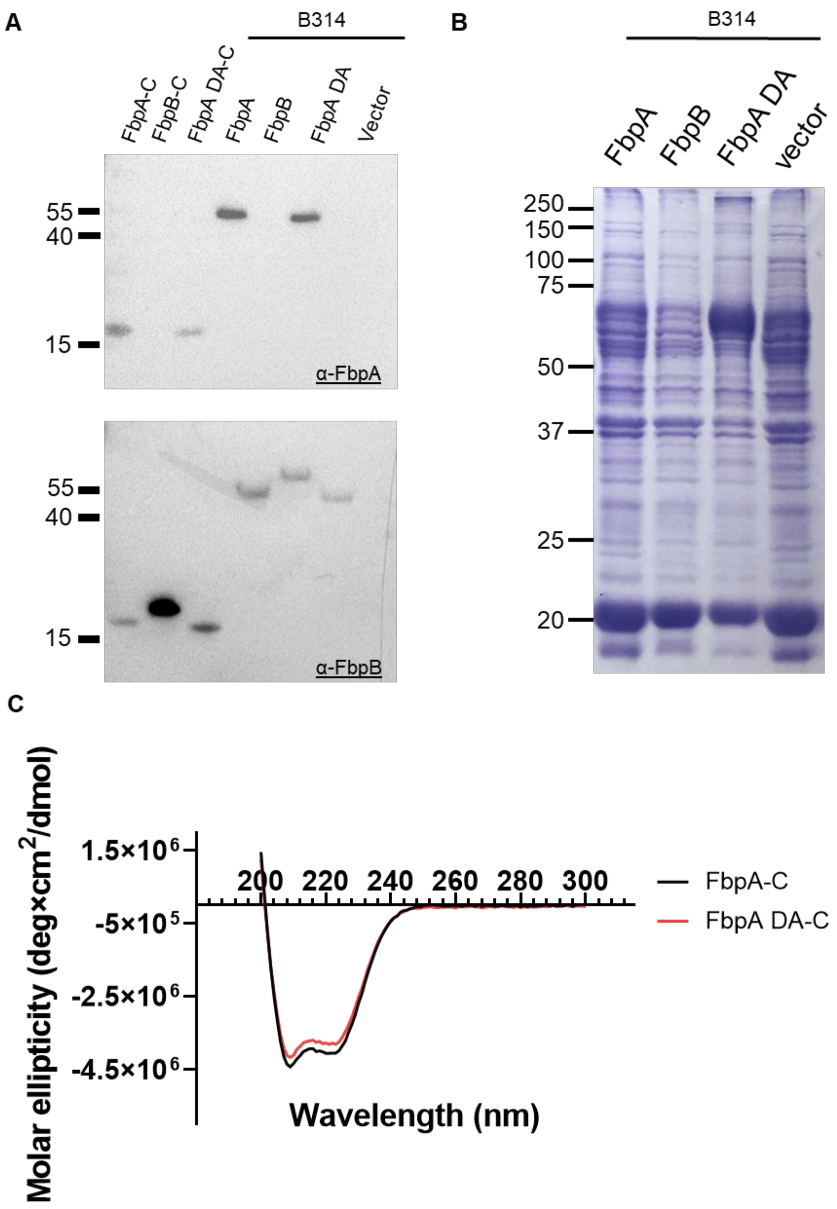

Supplemental Figure 2. *B. burgdorferi* B314 expression of *B. miyamotoi* FbpA and FbpB. (A) *B. burgdorferi* B314 strains were generated with ectopically expressed *B. miyamotoi* FbpA, FbpB, and FbpA-R264A-K343A (FbpA DA). 2.5 x10^7 whole cell equivalents were resolved by SDS-PAGE then transferred onto PVDF membranes for Western blotting. Membranes were then incubated with each anti-Fbp-specific mouse serum for FbpA (top) or FbpB (bottom) to determine expression levels. Recombinant FbpA-C, FbpB-C, and lysates from B314 pBBE22*luc* were used as positive and negative controls, respectively. Mouse anti-FbpB serum demonstrated cross-reactivity with FbpA. (B) 2.5 x10^7 whole cell equivalents were resolved by SDS-PAGE and served as a loading control for the Far Western. (C) Circular dichroism performed on both FbpA-C and FbpA DA-C indicates that both proteins are folded similarly and retain alpha helical structure.

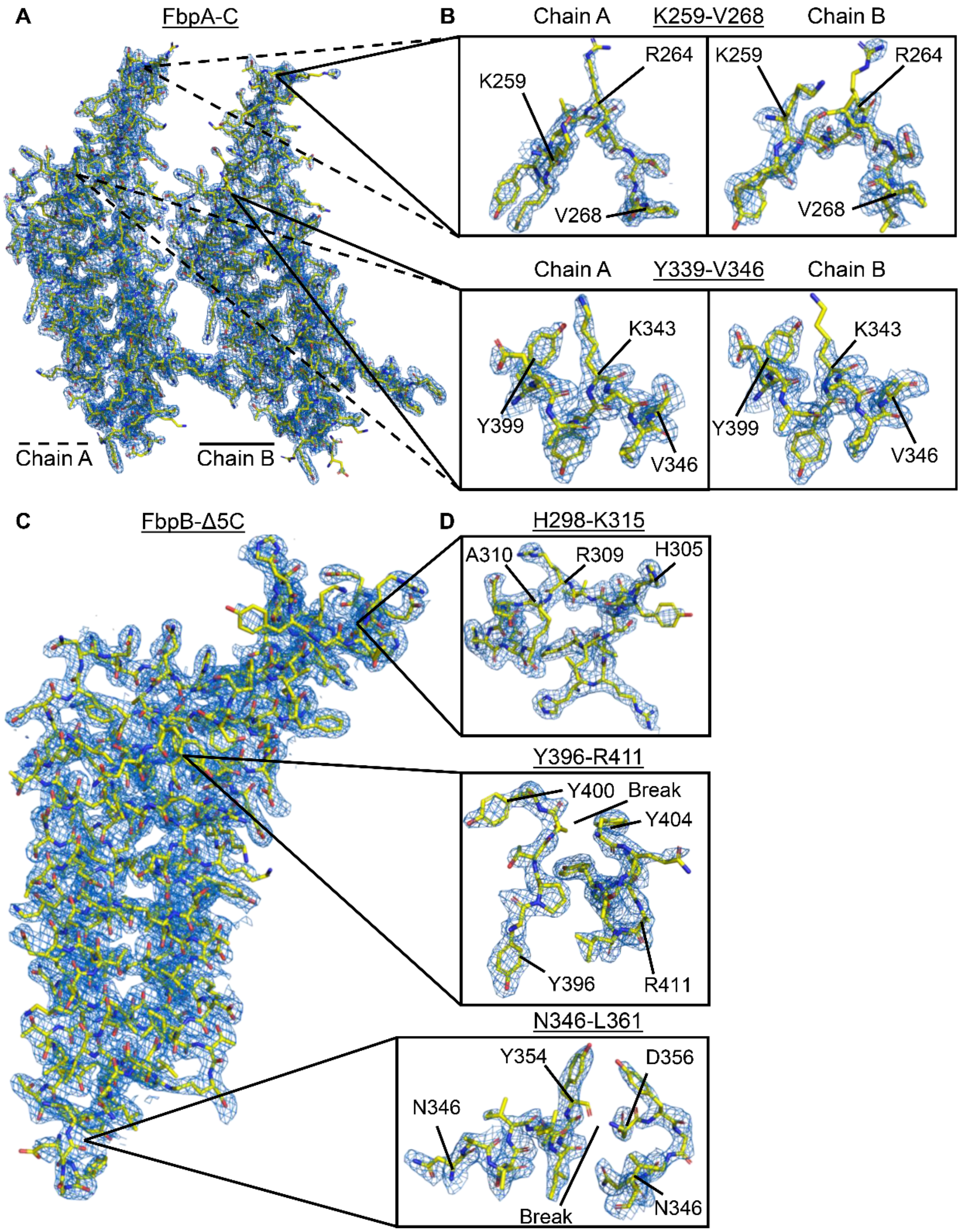

Supplemental Figure 3. Electron density maps for the refined FbpA-C and FbpBΔ5-C structures. (A) The final refined structure for FbpA-C with corresponding electron density for the areas surrounding R264 and K343 in (B). (C) The final refined structure for FbpB-Δ5C with corresponding electron density for the area surrounding R309 and places of chain break due to poor electron density (D). All models are shown as yellow sticks with a 2*Fo-Fc* map contoured at 1.2 σ around each polypeptide chain. FbpA-C contained two copies within the asymmetric unit which are labeled Chain A and Chain B whereas FbpB-Δ5C contained only one copy within the asymmetric unit denoted Chain A.

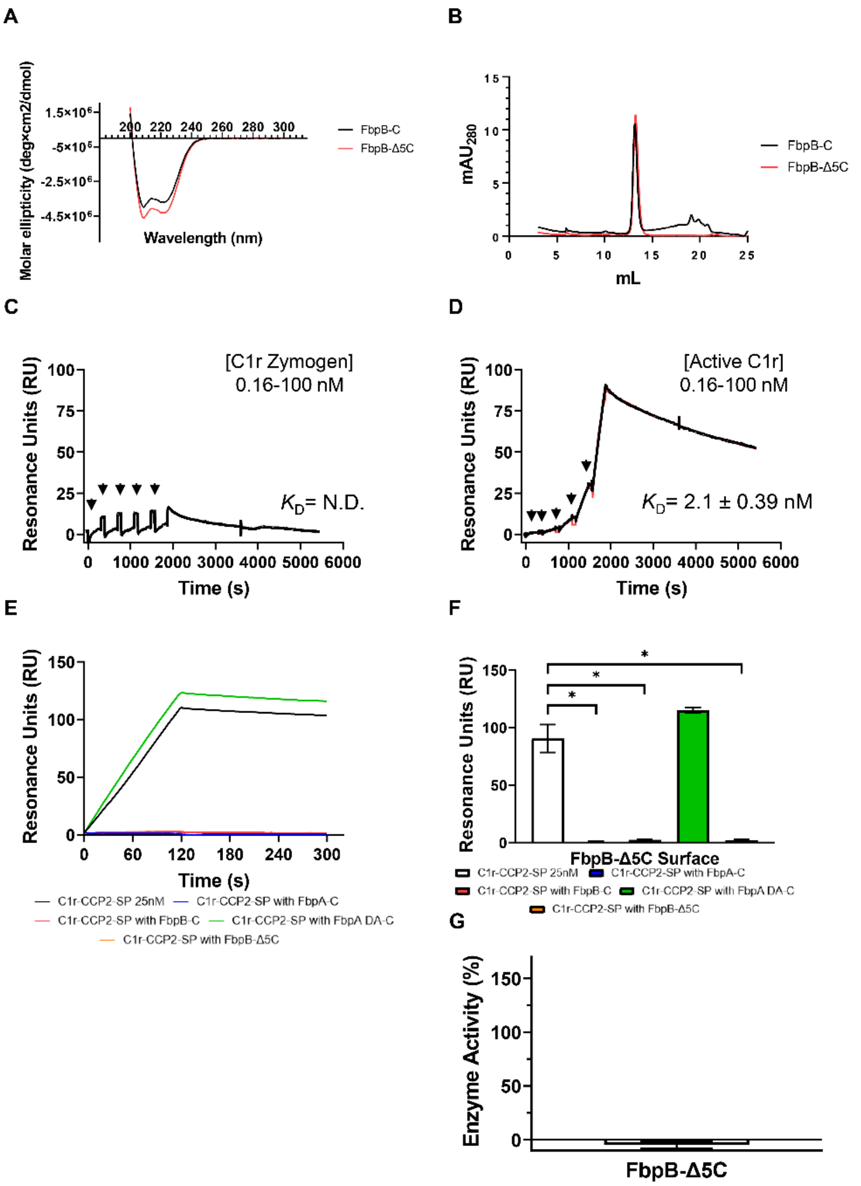

Supplemental Figure 4. Structural and biochemical properties of FbpB-Δ5C$_{267-427}$. (A) Circular dichroism was performed on both FbpB-C and FbpB-ΔC and indicates that both proteins retain similar alpha helical structure as indicated by negative peaks at 208 and 222 nm. (B) Analytical gel filtration was performed using Superdex 75 10/300 GL column. SPR was used to determine (C) C1r zymogen or (D) active C1r binding of the crystallographic construct FbpB-Δ5C. Binding affinities of full-length purified C1r in its zymogen and active enzymatic forms with FbpB-Δ5C were determined using the same methods as for the FbpB-C data shown in **Fig. 6A.** (E) Implementing the same SPR competition for C1r-CCP2-SP as in **Figs. 3D-E**, a FbpB-Δ5C immobilized surface was able to bind and compete for 25 nM C1r-CCP2-SP like FbpA-C and FbpB-C. (F) Max binding from each injection from (E). (G) 1 μM of FbpB-Δ5C was incubated with 15 nM C1r-CCP2-SP and then assessed for C1r activity using the colorimetric substrate Z-Gly-Arg. FbpB-Δ5C retained similar inhibitory activities as FbpB-C shown in **Fig. 6D**. One-way ANOVA followed by multiple comparison Tukey test determined statistical differences ($p<0.05$).

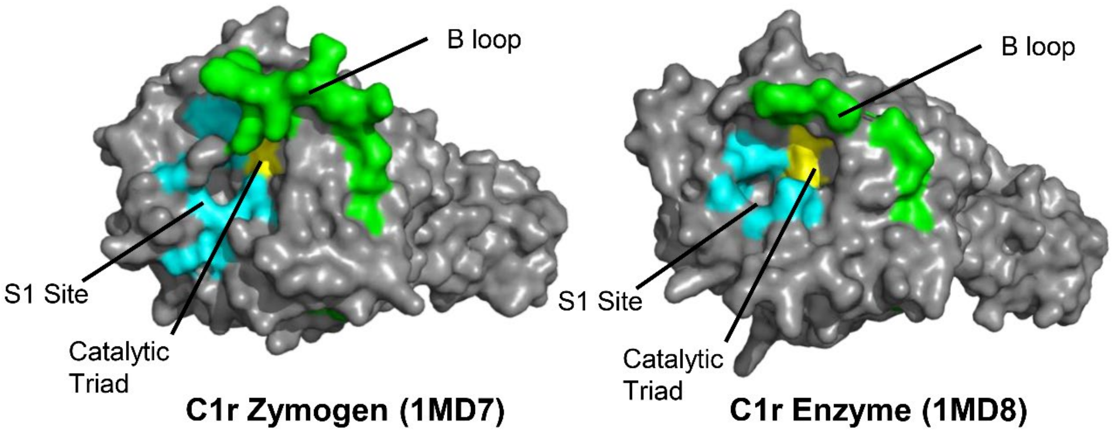

Supplemental Figure 5. Structural differences of C1r active states. Zymogen (PDB: 1MD7) and enzymatic (PDB: 1MD8) C1r-CCP2-SP structures highlighting key differences are shown. The B-loop (green) occludes the S1 site (cyan) and catalytic triad (yellow) in the zymogen state, whereas in the active state the B-loop adopts a conformation that presumably allows access to the newly formed S1 site (cyan) and catalytic triad (yellow).
